# Supplementary material for: A Vancomycin HPLC Assay for Use in Gut Microbiome Research
Source: Microbiol Spectr. 2022 May 10;10(3):e01688-21. doi: 10.1128/spectrum.01688-21 (PMC9241942; doi:10.1128/spectrum.01688-21)
Supplement: SUPPLEMENTAL FILE 1 — Supplemental material. Download spectrum.01688-21-s001.pdf, PDF file, 0.4 MB [file spectrum.01688-21-s001.pdf]

Supplemental Materials

A Vancomycin HPLC Assay for Use in Gut Microbiome Research

Chenlin Hu, Nicholas D Beyda, Kevin W Garey*

University of Houston College of Pharmacy, Houston, Texas, USA

***Corresponding Author:**

Kevin W. Garey, PharmD, MS, FASHP, FIDSA

Department of Pharmacy Practice and Translational Research,

University of Houston College of Pharmacy, 4849 Calhoun Road, Houston, TX 77204

Phone: 832-842-8386; E-mail: kgarey@uh.edu

Content:

Table S1

Figure S1-S9

Table S1. Comparison of fecal vancomycin concentrations measured by the present method and previously published method by Berthoin et al (2009)

Sample	Fecal vancomycin concentration (Mean ± SD, μg/ml)		Ratio between present and previous method
	Present method	Previous method	
5	262±6	267±45	0.98
6	1,852±187	2,045±100	0.91
8	1,914±43	2,110±23	0.91
9	1,945±42	2,106±33	0.92
12	1,626±255	1,771±11	0.92
14	1,148±72	1,253±47	0.92

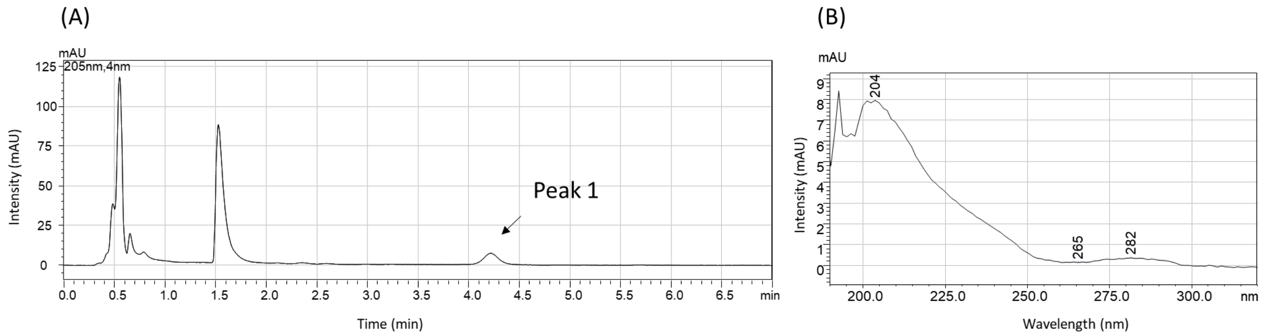

Figure S1: (A) The representative chromatographic profile of the fecal extract of the original clinical sample 5 collected from subject H on day 5, which was monitored at 205 nm. The Peak1 was identified to be the vancomycin. (B) The UV absorption pattern of Peak 1 (identified as vancomycin) that was consistent with that of standard vancomycin.

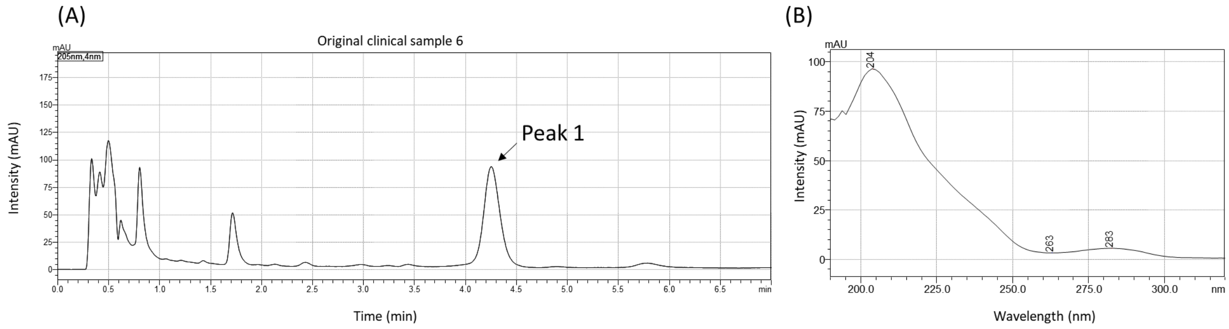

Figure S2: (A) The representative chromatographic profile of the fecal extract of the original clinical sample 6 collected from subject E on day 8, which was monitored at 205 nm. The Peak1 was identified to be the vancomycin. (B) The UV absorption pattern of Peak 1 (identified as vancomycin) that was consistent with that of standard vancomycin.

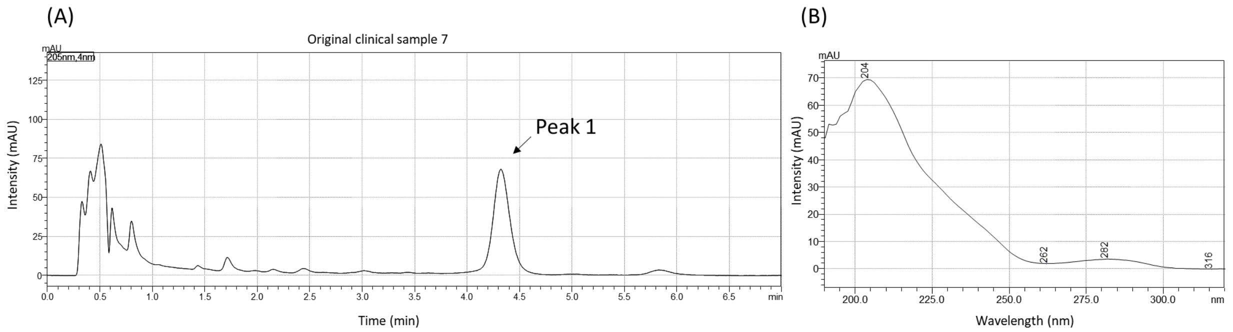

Figure S3: (A) The representative chromatographic profile of the fecal extract of the original clinical sample 7 collected from subject F on day 8, which was monitored at 205 nm. The Peak1 was identified to be the vancomycin. (B) The UV absorption pattern of Peak 1 (identified as vancomycin) that was consistent with that of standard vancomycin.

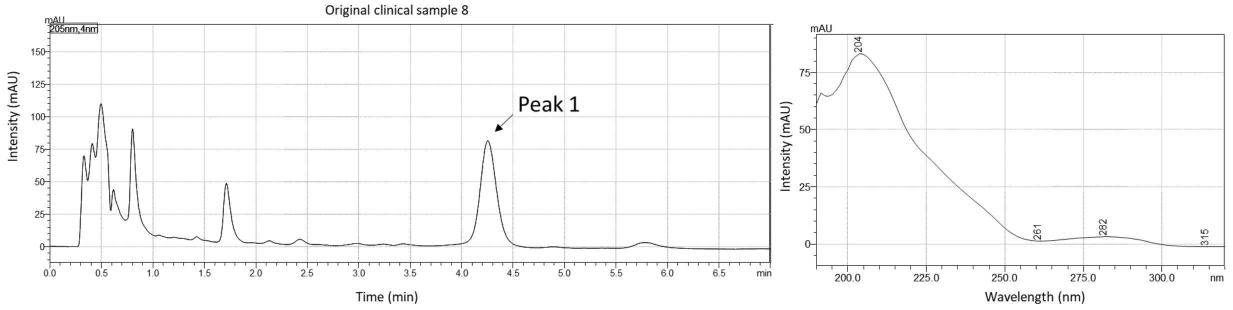

Figure S4: (A) The representative chromatographic profile of the fecal extract of the original clinical sample 8 collected from subject E on day 9, which was monitored at 205 nm. The Peak1 was identified to be the vancomycin. (B) The UV absorption pattern of Peak 1 (identified as vancomycin) that was consistent with that of standard vancomycin.

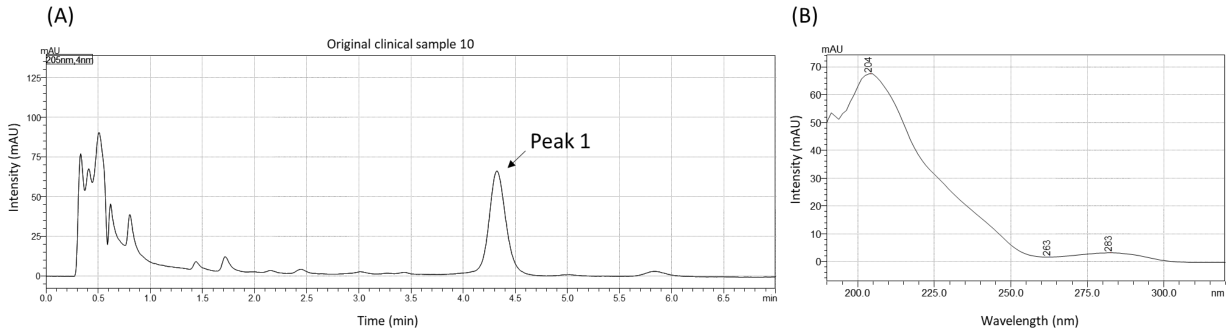

Figure S5: (A) The representative chromatographic profile of the fecal extract of the original clinical sample 10 collected from subject D on day 10, which was monitored at 205 nm. The Peak1 was identified to be the vancomycin. (B) The UV absorption pattern of Peak 1 (identified as vancomycin) that was consistent with that of standard vancomycin.

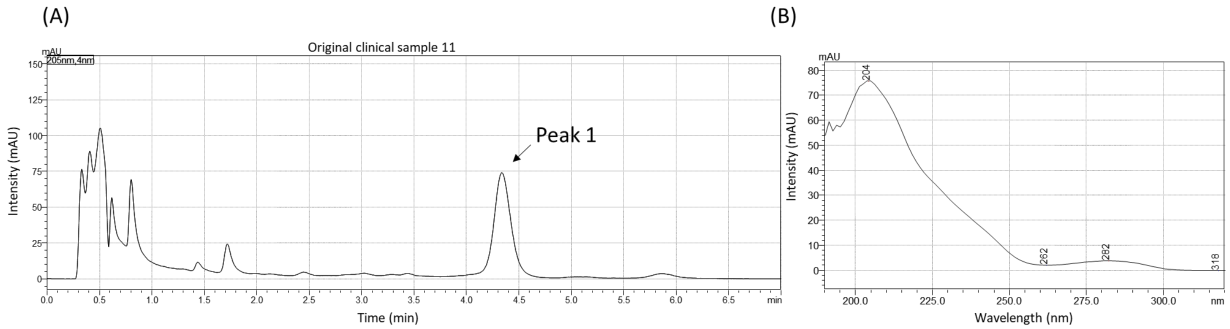

Figure S6: (A) The representative chromatographic profile of the fecal extract of the original clinical sample 11 collected from subject D on day 11, which was monitored at 205 nm. The Peak1 was identified to be the vancomycin. (B) The UV absorption pattern of Peak 1 (identified as vancomycin) that was consistent with that of standard vancomycin.

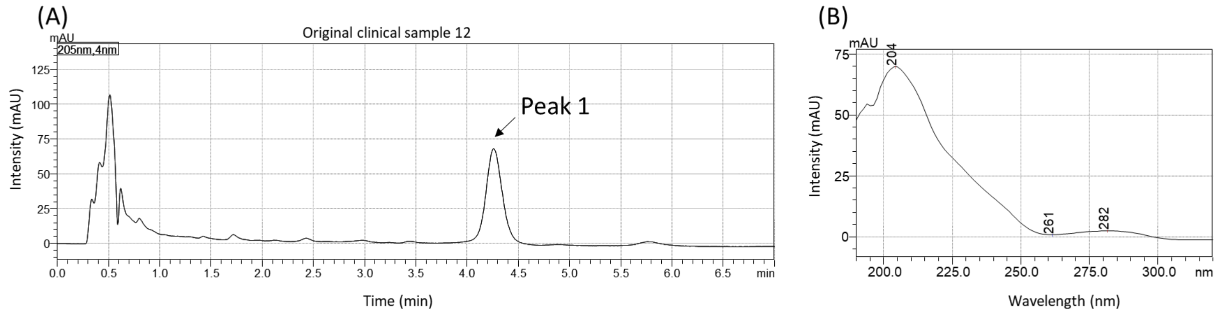

Figure S7: (A) The representative chromatographic profile of the fecal extract of the original clinical sample 12 collected from subject F on day 11, which was monitored at 205 nm. The Peak1 was identified to be the vancomycin. (B) The UV absorption pattern of Peak 1 (identified as vancomycin) that was consistent with that of standard vancomycin.

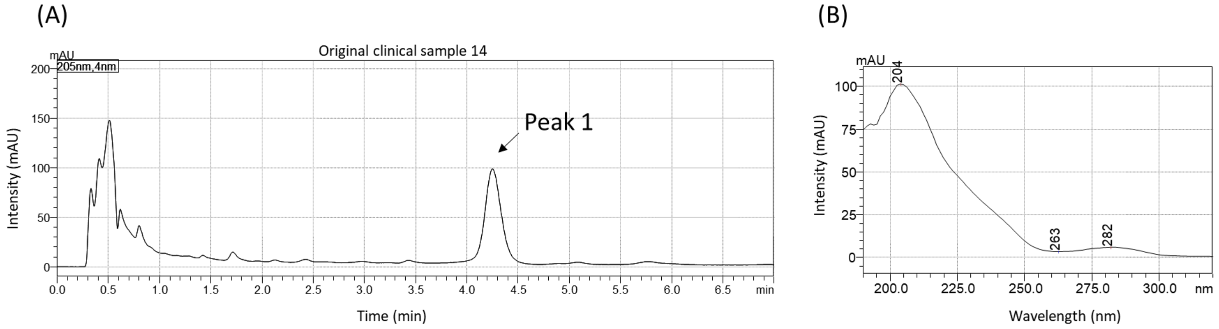

Figure S8: (A) The representative chromatographic profile of the fecal extract of the original clinical sample 14 collected from subject F on day 12, which was monitored at 205 nm. The Peak1 was identified to be the vancomycin. (B) The UV absorption pattern of Peak 1 (identified as vancomycin) that was consistent with that of standard vancomycin.

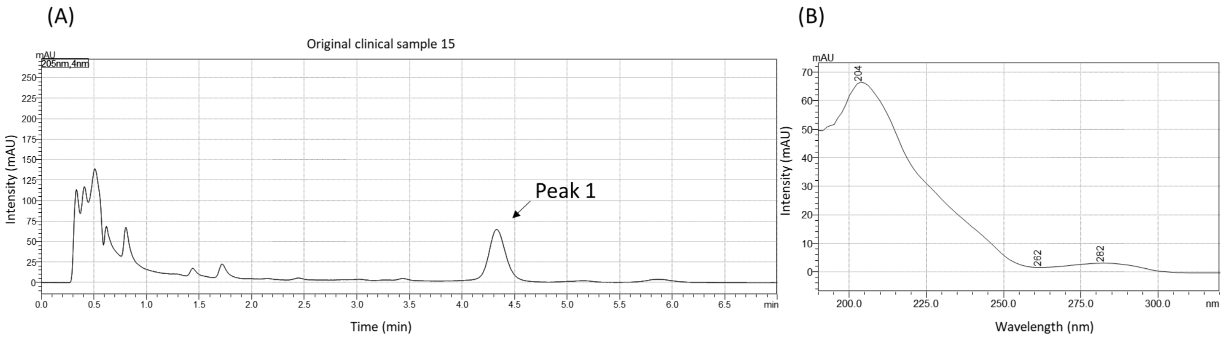

Figure S9: (A) The representative chromatographic profile of the fecal extract of the original clinical sample 15 collected from subject D on day 12, which was monitored at 205 nm. The Peak1 was identified to be the vancomycin. (B) The UV absorption pattern of Peak 1 (identified as vancomycin) that was consistent with that of standard vancomycin.
